# Supplementary material for: UV-LED Combined with Small Bioreactor Platform (SBP) for Degradation of 17α-Ethynylestradiol (EE2) at Very Short Hydraulic Retention Time
Source: Materials (Basel). 2021 Oct 11;14(20):5960. doi: 10.3390/ma14205960 (PMC8538786; doi:10.3390/ma14205960)
Supplement: Supplementary file 1 [file materials-14-05960-s001.zip › materials-1345014-supplementary.pdf]

Supporting Information

UV-LED Combined with Small Bioreactor Platform (SBP) for Degradation of 17α-Ethynylestradiol (EE2) at Very Short Hydraulic Retention Time

Oran Fradkin [1,2], Hadas Mamane [1], Aviv Kaplan [2], Ofir Menashe [3,4], Eyal Kurzbaum [5], Yifaat Betzalel [1] and Dror Avisar [2,*]

[1] School of Mechanical Engineering, Faculty of Engineering, Tel Aviv University, Tel Aviv 69978, Israel; oranfradkin20@gmail.com (O.F.); hadasmg@tauex.tau.ac.il (H.M.); yif.bet@gmail.com (Y.B.)
[2] The Hydrochemistry Laboratory, The Water Research Center, Porter School of the Environment and Earth Sciences, Faculty of Exact Sciences, Tel Aviv University, Tel Aviv 69978, Israel; avivkaplan@tauex.tau.ac.il
[3] Water Industry Engineering Department, Achi Racov Engineering School, Kinneret College on the Sea of Galilee, M.P. Emek Ha'Yarden 15132, Israel; ofirmn@mx.kinneret.ac.il
[4] BioCastle Water Technologies Ltd., Tzemach Industries Central Area, Jordan Valley 1510501, Israel
[5] Shamir Research Institute, University of Haifa, P.O. Box 97, Qatzrin 1290000, Israel; eyal3056@gmail.com
* Correspondence: droravi@tauex.tau.ac.il

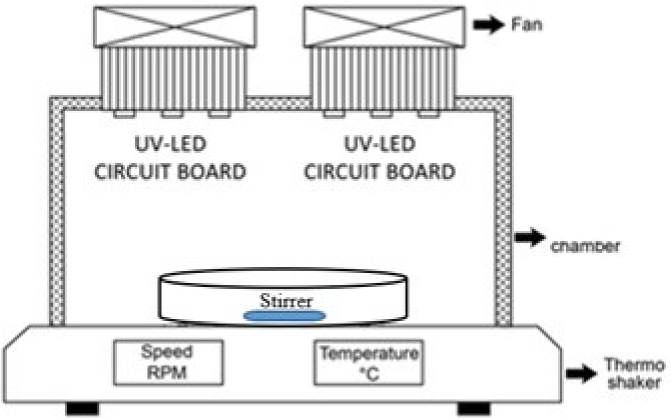

Figure S1. UV-LED rectangular system with a crystallization dish setup. Chamber configuration includes a thermo-shaker and crystallization dish.

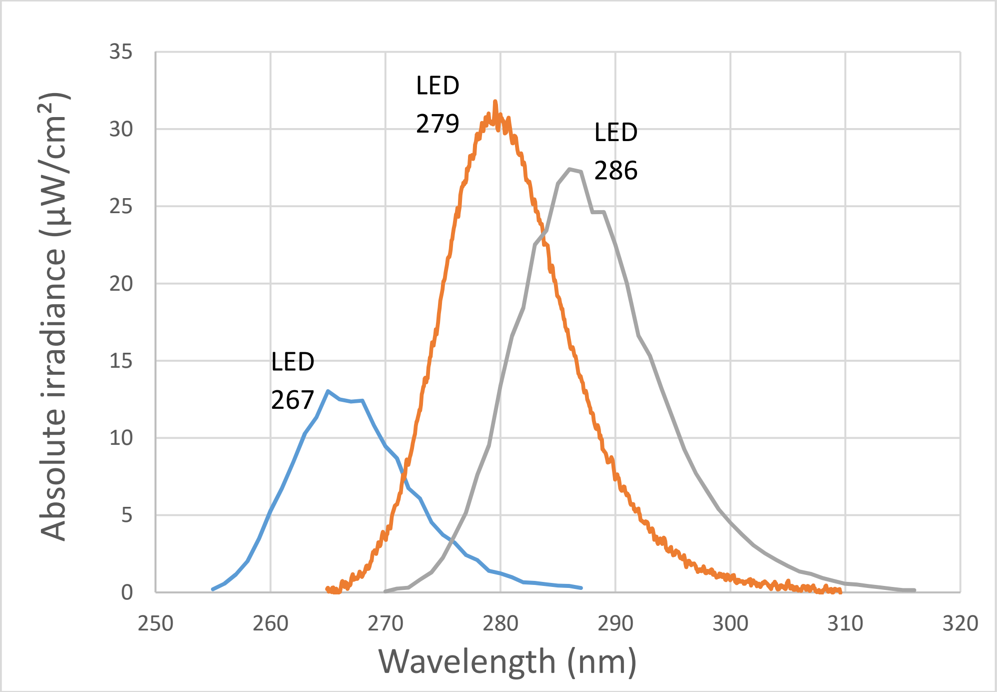

Figure S2. Emission spectra of the UV-LEDs for the circular and rectangular systems that used in this study.

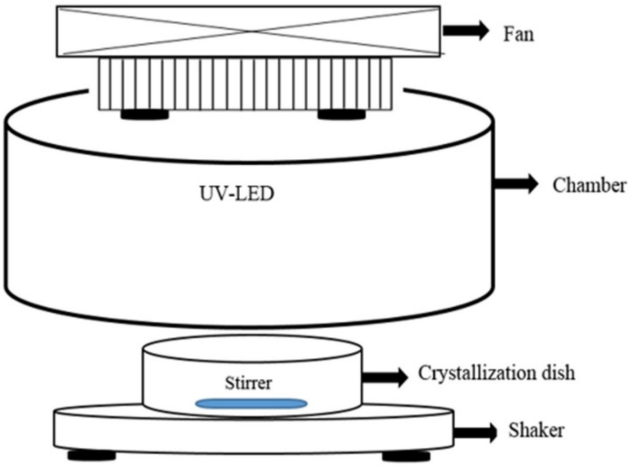

Figure S3. UV-LED circular system with a crystallization dish setup. Chamber configuration includes a shaker and crystallization dish. The configuration of the nine UV-LEDs is circular.

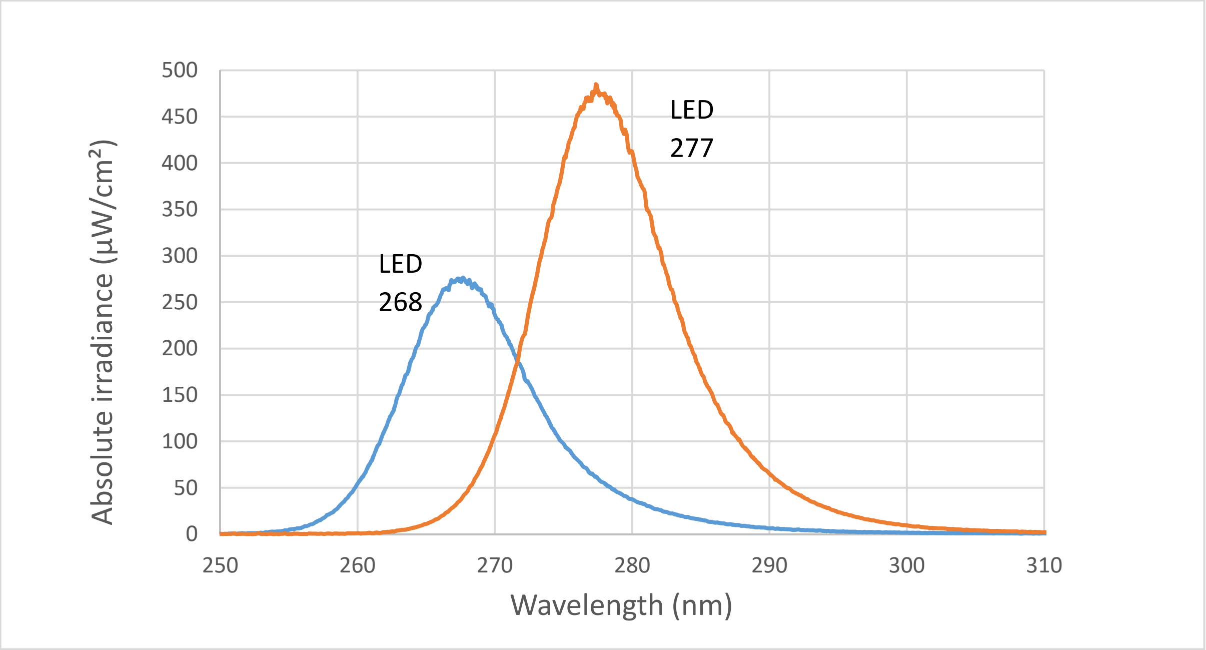

Figure S4. Emission spectra of the UV-LEDs for the OBR that used in this study.

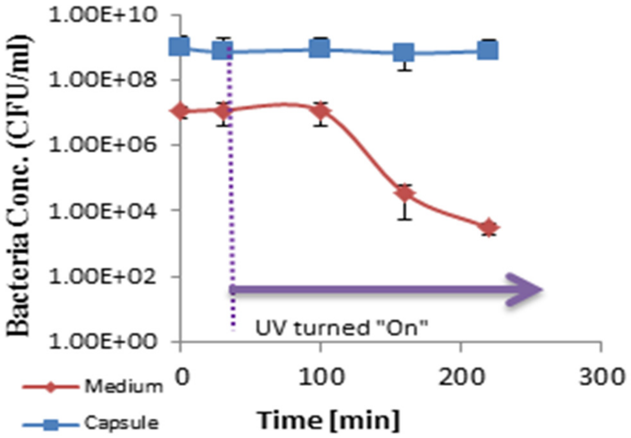

Figure S5. UV irradiation effect on the suspended (within the medium-control) and encapsulated (within the SBP capsule) bacteria.

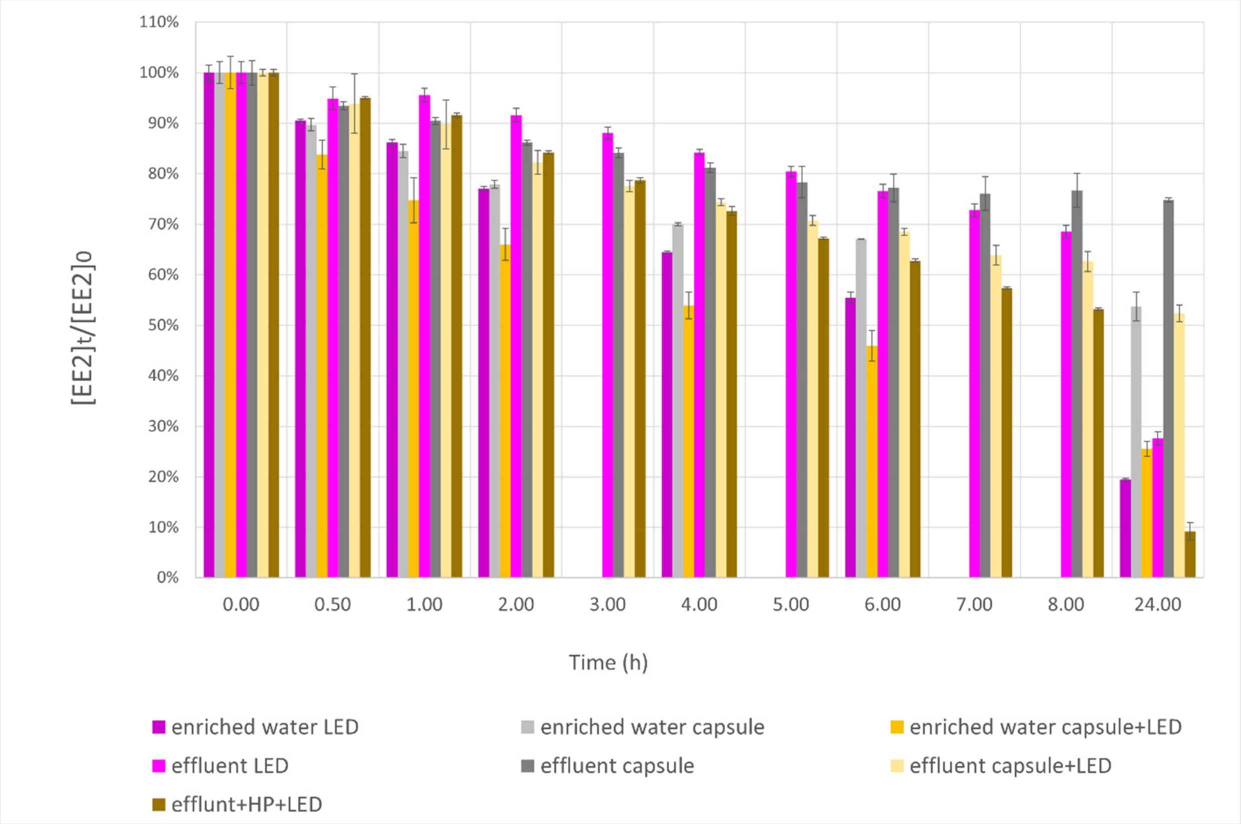

Figure S6. Comparison of different processes: UV-LEDs wavelengths (267.7 nm and 277.4 nm), SBP (encapsulate Rhodococcus zopfii culture) and both together. In two different mediums: water enriched, and effluents WW. Furthermore, UV-LEDs with (10 mg/L) H_2O_2, all in 3L versus time (hours).

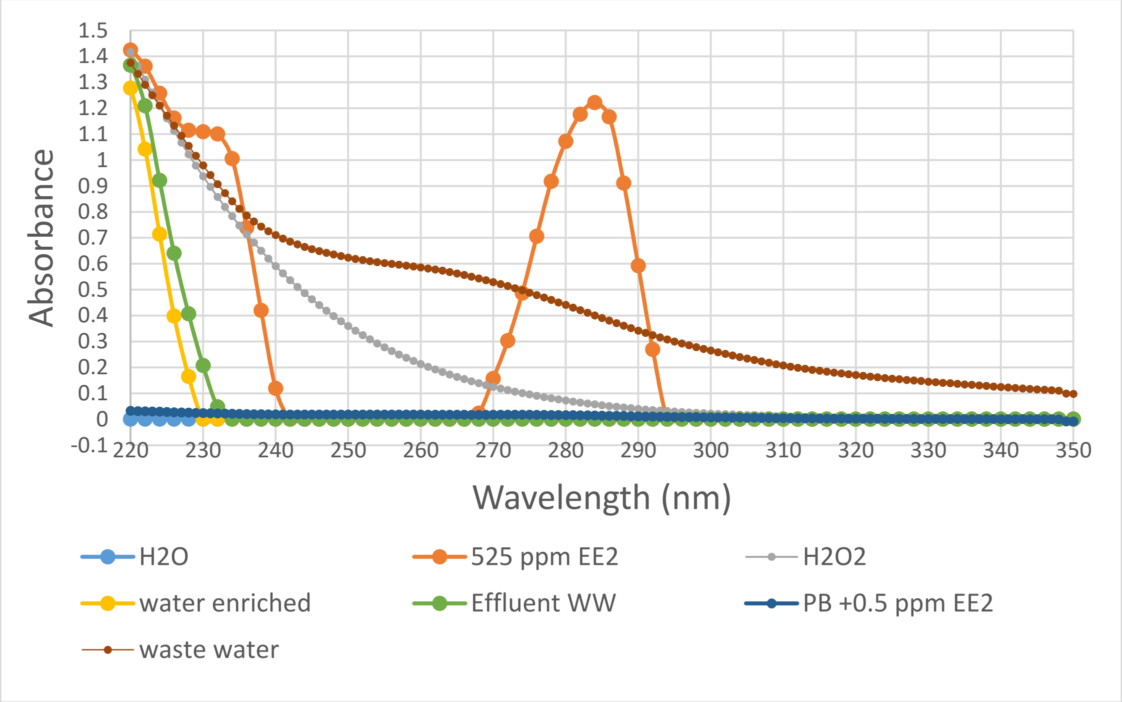

Figure S7. The solutions background absorbance for H_2O_2, PB+ 0.5 EE2, enriched water and effluent. Additionally, EE2 show maximum absorption at 284 nm and another peak at 220 nm. The H_2O_2 absorbance was the highest at 220 nm and decline towards 300 nm.

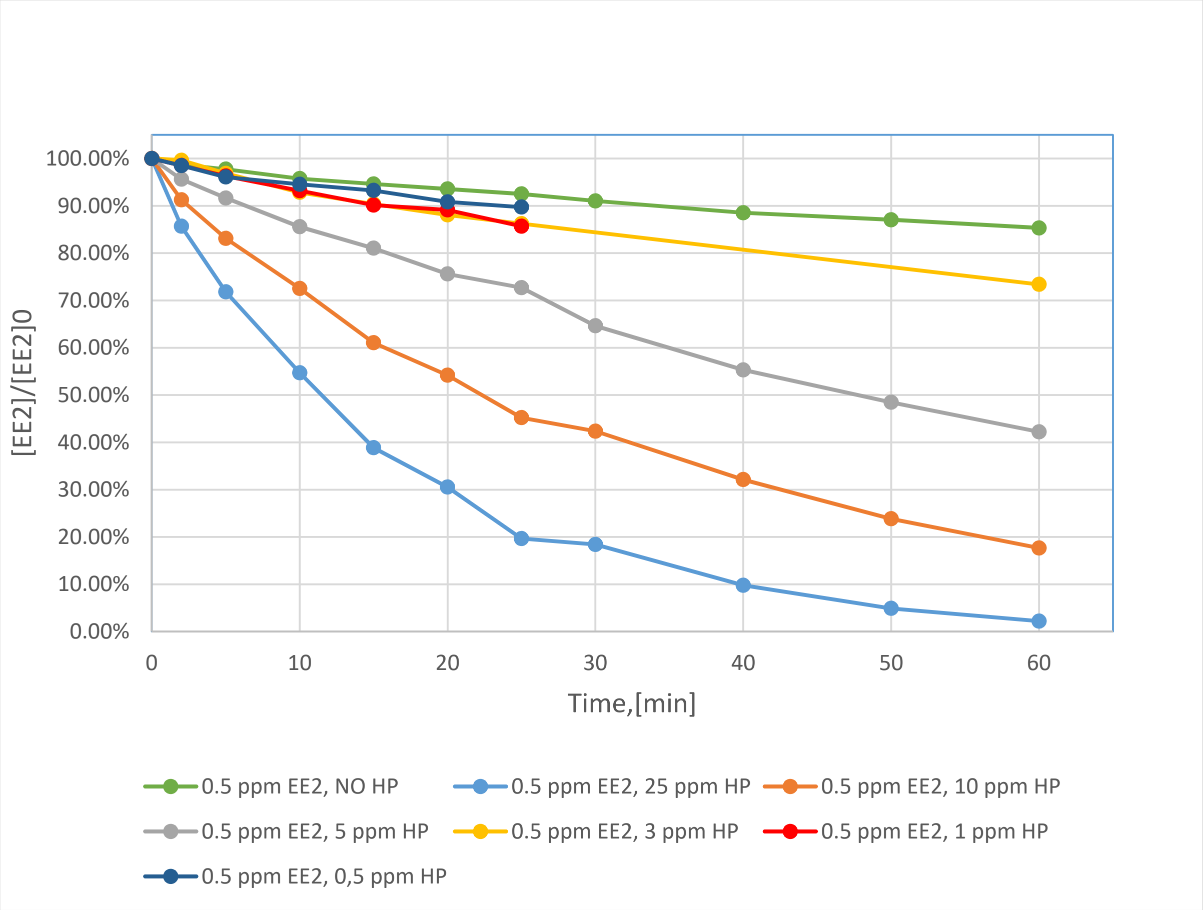

Figure S8. Degradation of 0.5 mg/L EE2 by direct UV-LED, using various concentrations ranging from 0 to 25 mg/L of H_2O_2 HP, with LED wavelength 267.2 nm versus time (minutes).

In general, higher peroxide concentration results in higher EE2 removal, hence, peroxide concentration governs the extent of EE2 degradation. As shown, the EE2 removal is negligible below 5 mg/L of peroxide. Finally, we repeated all the section to get trusted values and standard deviation, that was not more than 2.5%. The concentration of peroxide was: no, 0.5, 1, 3, 5, 10, 25 ppm. After determining 10 mg/L of peroxide as the optimal concentration for EE2 AOP treatment, due to, it was the first concentration that degraded EE2 by 80% in less than hour.

Link for bio-castel, that shows how Oran Fradkin is making capsule: https://www.youtube.com/watch?app=desktop&v=yEMjx2FZT5Y (accessed on 11 October 2021).
